# Association of leukocyte DNA methylation changes with dietary folate and alcohol intake in the EPIC study

**DOI:** 10.1186/s13148-019-0637-x

**Published:** 2019-04-02

**Authors:** F. Perrier, V. Viallon, S. Ambatipudi, A. Ghantous, C. Cuenin, H. Hernandez-Vargas, V. Chajès, L. Baglietto, M. Matejcic, H. Moreno-Macias, T. Kühn, H. Boeing, A. Karakatsani, A. Kotanidou, A. Trichopoulou, S. Sieri, S. Panico, F. Fasanelli, M. Dolle, C. Onland-Moret, I. Sluijs, E. Weiderpass, J. R. Quirós, A. Agudo, J. M. Huerta, E. Ardanaz, M. Dorronsoro, T. Y. N. Tong, K. Tsilidis, E. Riboli, M. J. Gunter, Z. Herceg, P. Ferrari, I. Romieu

**Affiliations:** 1 Nutritional Methodology and Biostatistics Group, International Agency for Research on Cancer (IARC), World Health Organization, 150, cours Albert Thomas, 69372 Lyon CEDEX 08, France; 2 Epigenetics Group, IARC, Lyon, France; 3 MRC Integrative Epidemiology Unit, Bristol Medical School, University of Bristol, Bristol, UK; 4 Nutritional Epidemiology Group, IARC, Lyon, France; 5 Department of Clinical and Experimental Medicine, University of Pisa, Pisa, Italy; 6 Department of Preventive Medicine, Keck School of Medicine, University of Southern California/Norris Comprehensive Cancer Center, Los Angeles, CA USA; 7 Universidad Autonoma Metropolitana, Mexico City, Mexico; 8 Division of Cancer Epidemiology, German Cancer Research Center (DKFZ), Heidelberg, Germany; 9 Department of Epidemiology, German Institute of Human Nutrition (DIfE), Potsdam-Rehbrücke, Germany; 10 Hellenic Health Foundation, Athens, Greece; 11 2nd Pulmonary Medicine Department, School of Medicine, National and Kapodistrian University of Athens, “ATTIKON” University Hospital, Haidari, Greece; 12 1st Department of Critical Care Medicine and Pulmonary Services, University of Athens Medical School, Evangelismos Hospital, Athens, Greece; 13 Epidemiology and Prevention Unit, Fondazione IRCCS Istituto Nazionale dei Tumori, Milan, Italy; 14 Dipartimento di Medicina Clinica e Chirurgia, Federico II University, Naples, Italy; 15 Cancer Epidemiology Unit, Department of Medical Sciences, University of Turin, Via Santena 7, Turin, Italy; 16 National Institute of Public Health and the Environment (RIVM), Centre for Health Protection (pb12), Bilthoven, The Netherlands; 17 Department of Epidemiology, Julius Center Research Program Cardiovascular Epidemiology, Utrecht, The Netherlands; 18 Department of Research, Cancer Registry of Norway, Institute of Population-Based Cancer Research, Oslo, Norway; 19 Department of Medical Epidemiology and Biostatistics, Karolinska Institutet, Stockholm, Sweden; 20 Genetic Epidemiology Group, Folkhälsan Research Center and Faculty of Medicine, University of Helsinki, Helsinki, Finland; 21 Department of Community Medicine, University of Tromsø, The Arctic University of Norway, Tromsø, Norway; 22 Public Health Directorate, Asturias, Spain; 23 Unit of Nutrition and Cancer, Cancer Epidemiology Research Program, Catalan Institute of Oncology-IDIBELL, L’Hospitalet de Llobregat, Barcelona, Spain; 24 Department of Epidemiology, Murcia Regional Health Council, IMIB-Arrixaca, Murcia, Spain; 25 CIBER Epidemiology and Public Health CIBERESP, Madrid, Spain; 26 Navarra Public Health Institute, Pamplona, Spain; 27 IdiSNA, Navarra Institute for Health Research, Pamplona, Spain; 28 Public Health Direction and Biodonostia Research Institute and CIBERESP, Basque Regional Health Department, San Sebastian, Spain; 29 Cancer Epidemiology Unit, Nuffield Department of Population Health, University of Oxford, Oxford, UK; 30 Department of Epidemiology and Biostatistics, School of Public Health, Imperial College London, London, UK

**Keywords:** DNA methylation, Dietary folate, Alcohol intake, DMR, Fused lasso, EPIC cohort

## Abstract

**Background:**

There is increasing evidence that folate, an important component of one-carbon metabolism, modulates the epigenome. Alcohol, which can disrupt folate absorption, is also known to affect the epigenome. We investigated the association of dietary folate and alcohol intake on leukocyte DNA methylation levels in the European Prospective Investigation into Cancer and Nutrition (EPIC) study. Leukocyte genome-wide DNA methylation profiles on approximately 450,000 CpG sites were acquired with Illumina HumanMethylation 450K BeadChip measured among 450 women control participants of a case-control study on breast cancer nested within the EPIC cohort. After data preprocessing using surrogate variable analysis to reduce systematic variation, associations of DNA methylation with dietary folate and alcohol intake, assessed with dietary questionnaires, were investigated using CpG site-specific linear models. Specific regions of the methylome were explored using differentially methylated region (DMR) analysis and fused lasso (FL) regressions. The DMR analysis combined results from the feature-specific analysis for a specific chromosome and using distances between features as weights whereas FL regression combined two penalties to encourage sparsity of single features and the difference between two consecutive features.

**Results:**

After correction for multiple testing, intake of dietary folate was not associated with methylation level at any DNA methylation site, while weak associations were observed between alcohol intake and methylation level at CpG sites cg03199996 and cg07382687, with *q*_val_ = 0.029 and *q*_val_ = 0.048, respectively. Interestingly, the DMR analysis revealed a total of 24 and 90 regions associated with dietary folate and alcohol, respectively. For alcohol intake, 6 of the 15 most significant DMRs were identified through FL.

**Conclusions:**

Alcohol intake was associated with methylation levels at two CpG sites. Evidence from DMR and FL analyses indicated that dietary folate and alcohol intake may be associated with genomic regions with tumor suppressor activity such as the *GSDMD* and *HOXA5* genes. These results were in line with the hypothesis that epigenetic mechanisms play a role in the association between folate and alcohol, although further studies are warranted to clarify the importance of these mechanisms in cancer.

**Electronic supplementary material:**

The online version of this article (10.1186/s13148-019-0637-x) contains supplementary material, which is available to authorized users.

## Introduction

DNA methylation is a crucial epigenetic mechanism involved in regulating important cellular processes, including gene expression, cell differentiation, genomic imprinting, and preservation of chromosome stability. DNA methylation refers to the addition of methyl groups (–CH3) to the carbon-5 position of cytosine residues in a cytosine-guanine DNA sequence (CpG) by DNA methyltransferases. DNA methylation changes can be influenced by many factors including aging [17, 19] and environmental exposure such as smoking [1, 24] or specific dietary factors [35]. Experimental evidence suggests a link between B vitamins, including folate (vitamin B_9_), and epigenetic modifications [3]. B vitamins, especially folate, are essential components of one-carbon metabolism (OCM), the network of interrelated biochemical reaction in which a one-carbon unit is received from methyl donor nutrients and transferred into biochemical and molecular pathways essential for DNA replication and repair. Modifications in OCM can significantly impact gene expression and thereby cellular function [53].

**Fig. 1:**
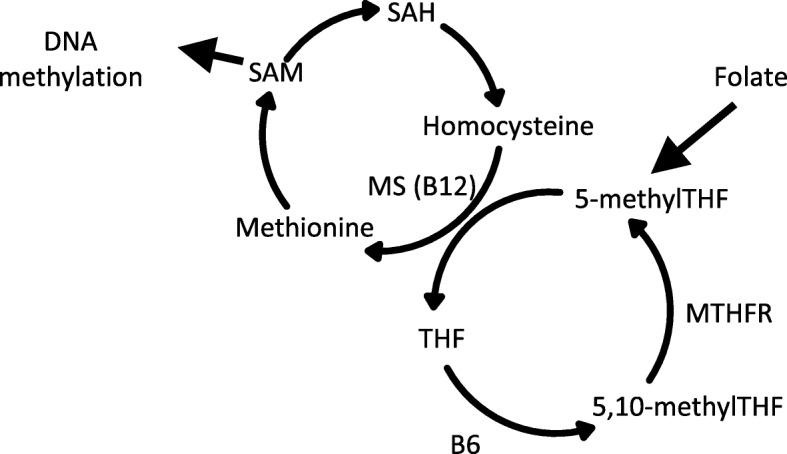
Diagram of the one-carbon metabolism pathway. MS methionine synthase, MTHFR methylenetetrahydrofolate reductase, THF tetrahydrofolate, SAH *S*-adenosylhomocysteine, SAM *S*-adenosylmethionine

Absorbed folate, circulating in the bloodstream, enters the OCM cycle in the liver where it is metabolized to 5-methyltetrahydrofolate (5-methylTHF) and converted into *S*-adenosylmethionine (SAM) after several successive transformation steps (Fig. 1). SAM is the methyl donor for numerous methylation reactions including the methylation of DNA, RNA, and proteins. The potential role of specific dietary factors including micronutrients such as folate, alcohol, and soya intake, in modifying breast cancer risk via epigenetic mechanisms, has been proposed [54], although evidence is still scarce and inconsistent.

Alcohol intake affects epigenetic profiles [32]. Ethanol metabolism generates toxins that may directly lead to OCM dysfunction by reducing folate absorption, increasing renal excretion of folate, and inhibiting methionine synthase, the key enzyme in the generation of the methyl donor in the OCM [32, 33]. This antagonistic effect of alcohol on folate could plausibly increase the need of folate intake. Inadequate folate levels may result in abnormal DNA synthesis due to a reduced availability of SAM [27] and disrupted DNA repair and may, hence, influence cancer risk, including breast cancer [4, 60].

The epidemiological evidence linking dietary folate, alcohol intake, and epigenome modifications is, however, not well documented. Therefore, we investigated the relationships between dietary folate and alcohol intake with leukocyte DNA methylation patterns in the controls from the European Prospective Investigation into Cancer and Nutrition (EPIC) study on breast cancer. We complemented standard regression analysis with techniques for the identification of relevant methylated regions.

## Methods

### Study population

EPIC is a multicenter study that recruited over 521,000 participants, between 1992 and 2000 in 23 regional or national centers in 10 European countries (Denmark, France, Germany, Greece, Italy, The Netherlands, Norway, Spain, Sweden, and the UK) [43]. Among the 367,903 women recruited in EPIC, and after exclusion of 19,583 participants with prevalent cancers at recruitment (except non-melanoma skin cancer), first malignant primary BC occurred for 10,713 women during follow-up between 1992 and 2010. Within a nested case-control study that included 2491 invasive BC cases [34], a subsample of 960 women who completed dietary and lifestyle questionnaires and provided blood samples at recruitment (480 cases and 480 matched controls) from Germany, Greece, Italy, The Netherlands, Spain, and the UK was selected for the DNA methylation analyses [2]. The present study included analysis of 450 controls only originally enrolled in this case-control study on breast cancer (BC) nested within the EPIC study.

### Methylation acquisition

Genome-wide DNA methylation profiles in buffy coat samples were quantified using the Illumina Infinium HumanMethylation 450K (HM450K) BeadChip assay [5] in 960 biospecimens from women included in the BC nested case-control study. A total of 20 biospecimens with replicates used to compare technical inter- and intra-assay batch effects and then excluded from the main analysis together with 19 matched pairs, i.e., 38 samples, where at least one of the two samples had a low-quality bisulfite conversion efficiency (intensity signal< 4000) or did not pass all of the Illumina GenomeStudio quality control steps, which were based on built-in control probes for staining, hybridization, extension, and specificity [23]. To prevent collider bias [11], as both alcohol intake and folate intake and DNA methylation profiles are all potentially associated with causes of BC, among the 902 remaining samples from the original case-control study on BC nested within EPIC study, only cancer-free women were selected for the present study. For the 451 controls sample, probes with detection *p* values higher than 0.05 were assigned “missing” value. After the exclusion of 14,548 cross-reactive probes [10], 47,963 probes overlapping known SNPs with minor allele frequency (MAF) greater than 5% in the overall population (European ancestry) [10] and 1483 low-quality probes (i.e., missing in more than 5% of the samples), 421,583 probes were left for the statistical analyses [2].

For each probe, *β* values were calculated as the ratio of methylated intensity over the overall intensity, defined as the sum of methylated and unmethylated intensities. The following preliminary adjustment steps were applied to *β* values: (i) color bias normalization using smooth quantile normalization [13], (ii) quantile normalization [6], and (iii) type I and type II bias correction using the beta-mixture quantile normalization (BMIQ) [56]. Then, *M* values, defined as ${M}_{\mathrm{values}}={\log}_2\left(\frac{\beta_{\mathrm{values}}}{1-{\beta}_{\mathrm{values}}}\right)$, were computed [14]. Surrogate variable analysis (SVA) [30, 31] was used to remove systematic variation due to the processing of the biospecimens during methylation acquisition such as batch, indicating groups of samples processed at the same time, and the position of the samples within the chip [40]. Then *M* values were standardized to have an identical variance of 1.

The percentage of white blood cell counts, i.e., T cells (CD8^+^T and CD4^+^T), natural killer (NK) cells, B cells, monocytes, and granulocytes, was quantified using Houseman’s estimation method [20] and included as covariates in the analysis.

### Lifestyle and dietary exposures

Data on dietary habits were collected at recruitment through validated center- or country-specific dietary questionnaires (DQ) [43]. Northern Italy (Florence, Turin, and Varese), UK, Germany, and The Netherlands used self-administered extensive quantitative food-frequency questionnaires (FFQs), whereas Southern Italy (Naples and Ragusa), Spain, and Greece’s centers used interview methods. Usual consumption of alcoholic beverages (number of glasses per day or week) per type of alcoholic beverage (wine, beer, spirits, and liquors) during the 12 months before the administration of dietary questionnaires was collected at recruitment. In addition, 24-h dietary recall (R) harmonized across EPIC countries was collected from a random sample (*n* = 36,900) in each center to be used as reference measurements [50]. R measurements were used to improve estimation of alcohol content per specific alcoholic beverages using a country-specific estimation of average of glass volume [48]. Dietary folate intake (μg/day) was estimated using the updated EPIC Nutrient Data Base (ENDB) [49], obtained after harmonization from country-specific food composition tables [7]. No specific information on the use of folate supplements was available.

### Statistical analyses

After exclusion of one outlier value of dietary folate (value larger than the third quartile plus 10 times the inter-quartile range of the distribution), a total of 450 observations from controls only were retained for statistical analyses.

The association between dietary folate, alcohol intake, and methylation levels was evaluated via (i) CpG site-specific analysis, (ii) identification of differentially methylated regions (DMRs) [41], and (iii) fused lasso (FL) regression [57].

### CpG site-specific models

*M* values expressing methylation levels at each CpG were linearly regressed on dietary folate (log-transformed to reduce skewness) and alcohol intake. Models were adjusted for recruitment center, age at recruitment (year), menopausal status (pre- or post-menopause), and white blood cell counts (proportions of T cells, natural killer cells, B cells, and monocytes in blood). False discovery rate (FDR) was used to control statistical tests for multiple testing.

For the two CpG sites that were associated with alcohol intake, based on *q* values, the percentage of methylation change for 1 standard deviation (SD) increase of alcohol intake was calculated as follows:

Methylation values in site *j* were log-transformed and regressed on alcohol intake (*A*_*i*_), for each site *j*, and for *i* = 1, … , *n*, as:

$$ \log \left({M}_{ij}\right)={\alpha}_{0j}+{\alpha}_{1j}{A}_i+{\gamma_j}^T{Z}_i $$

where *α*_1*j*_ estimate the regression coefficient, *Z*_*i*_ is a vector of confounding factors related to methylation levels through a vector of regression coefficients *γ*_*j*_. The ratio of any two log-transformed methylation values log(*M*_*ij*1_) and log(*M*_*ij*0_) with a difference of alcohol intake of 1 SD (${\widehat{\sigma}}_{\mathrm{alc}}$) was predicted as ${\widehat{\alpha}}_{1j}{\widehat{\sigma}}_{\mathrm{alc}}$. Therefore, the average percentage of methylation change for an increase of 1-SD in alcohol intake was estimated as:

$$ \frac{M_{ij1}}{M_{ij0}}=\left(\mathit{\exp}\left({\widehat{\alpha}}_{1j}{\widehat{\sigma}}_{\mathrm{alc}}\right)-1\right)\ast 100 $$

### DMR models

Differentially methylated region (DMRs) analyses were identified with the *DMRcate* package [41]. The rationale of this method is to use kernel smoothing to replace the *t* test statistics at a given CpG site by a weighted average of *t* test statistics across its neighboring sites on the same chromosome. More precisely, let *p*_*c*_ express the number of sites located on a given chromosome *c* with *c* ∈ {1,  … , 23} (the 23rd chromosome is chromosome X). For any site *k* on this chromosome, with *k* = 1, … , *p*_*c*_, the term *t*_*k*_^2^ indicates the square of the *t* test statistics obtained in site-specific analyses. For each site *j* on chromosome *c*, *t*_*j*_^2^ is replaced by the term ${{\widehat{t}}_j}^2$, defined as ${{\widehat{t}}_j}^2=\sum \limits_{k=1}^{p_c}{K}_{jk}{t_k}^{2.}$

where the terms *K*_*jk*_ express weights, with larger values for sites *k* closer to *j.* Let *x*_*k*_ express the position of site *k* on the chromosome, i.e., its chromosomal coordinate in base pairs, these weights are defined using a Gaussian kernel, as

$$ {K}_{jk}=\exp \left(\frac{-{\left|{x}_j-{x}_k\right|}^2}{2{\left(\lambda /C\right)}^2}\right) $$

where parameters *λ* and *C* represent the bandwidth and the scaling factor, respectively. Here, we used *λ* = 1000 and *C* = 2, respectively, as recommended in [41].

Under the null hypothesis of no association between site *j* and alcohol (or folate), the distribution of $\frac{{{\widehat{t}}_j}^2{\sum}_k^{p_c}{K}_{jk}}{\sum_k^n{K_{jk}}^2}$ can be approximated by a *χ*^2^ distribution [41] with ${\left({\sum}_k^{p_c}{K}_{jk}\right)}^2/{\sum}_k^{p_c}{K_{jk}}^2$ degrees of freedom [45]. Accordingly, *p* values were obtained for each site separately in each chromosome and *q* values were computed using FDR correction on all the *p* values to control for multiple testing. Then, DMRs were defined as regions with at least two significant sites separated by a maximal distance *λ* of 1000 base pairs. In line with [41], *t* statistics *t*_*k*_ were obtained from regression models using an empirical Bayes method to shrink the CpG site variance [51], as implemented in the *limma* package [52]. For each DMR, the minimum *q* value, the minimum and maximum coefficients (in absolute value) of the sites included in the region were presented as *q*_DMR_, *β*_min, DMR_, and *β*_max, DMR_.

### Fused lasso regression

Multivariate penalized regression provides an alternative to DMRs. We implemented a fused lasso (FL) regression [57], which is better suited than the standard lasso when covariates (CpGs) are naturally ordered and the objective is to identify regions on the chromosome of differentially methylated CpG sites. FL is particularly useful when the number of features (*p*) is way larger than the sample size (*n*), a situation classically known as *p* ≫ *n*.

FL is a multivariable regression method combining two penalties: (i) the lasso penalty, which introduces sparsity of the parameter vector, i.e., many elements of the estimated vector are encouraged to be set to zero, and (ii) the fused penalty, which encourages sparsity of the difference between two consecutive components in the parameter vector, thus introducing smoothness of parameter estimates in adjacent CpG sites [57].

To mimic the DMR analysis, a FL analysis was implemented where dietary folate and alcohol were, in turn, regressed on CpG methylation levels within each chromosome. The vector of methylation coefficient estimates $\widehat{\beta}$ obtained by fused lasso regression was defined as

$$ \widehat{\beta}=\arg \min \left\{{\sum}_i{\left({y}_i-{\sum}_j{M}_{ij}{\beta}_j-{\gamma}^T{Z}_i\right)}^2+{\widehat{\lambda}}_1{\sum}_{j=1}^{p_c}{\omega}_j\left|{\beta}_j\right|+{\widehat{\lambda}}_2{\sum}_{j=2}^{p_c}{\nu}_j\left|{\beta}_j-{\beta}_{j-1}\right|\right\}, $$

where *y*_*i*_ indicates, in turn, alcohol and dietary folate values for sample *i* = 1, … , *n*, *M*_*ij*_ is the methylation levels at CpG site *j*, *β*_*j*_ is the associated regression coefficient, *Z*_*i*_ is a vector of confounding factors, consistently with linear regression and DMR analyses described above, *γ* is the corresponding non-penalized vector of coefficients, and *ω*_*j*_ and *ν*_*j*_ are the weights associated with lasso penalty and fused penalty, respectively.

Following the rationale of the adaptive lasso [61] and the iterated lasso [8], the FL procedure was run for the first time with weights *ω*_*j*_ and *ν*_*j*_ set to 1, which returned ${\widehat{\beta}}_0$, an initial estimate of $\widehat{\beta}$. The final estimates $\widehat{\beta}$ were obtained after running a second FL procedure with weights defined as ${\omega}_j=\frac{1}{\left|{\widehat{\beta}}_{0,j}\right|+\varepsilon }$ and ${\nu}_j=\frac{1}{\left|{\widehat{\beta}}_{0,j}-{\widehat{\beta}}_{0,j-1}\ \right|+\varepsilon }$, with *ε* = 10^−4^.

The FL procedure was implemented on a predefined grid of 50 × 50 = 2500 values for the pair of parameters (*λ*_1_, *λ*_2_). More precisely, the grid for *λ*_1_ consisted of 50 equally spaced values (on a log scale) between $\frac{\lambda_{1,\max }}{1000}$ and *λ*_1, max_, where *λ*_1, max_ was the lowest *λ*_1_ value for which FL returned a null $\widehat{\beta}$ vector for *λ*_2_=0, a situation where FL reduces to a standard lasso. For each value *λ*_1_on this grid, the grid for *λ*_2_consisted of 50 equally spaced values (on a log scale) between $\frac{\lambda_{2,\max}\left({\lambda}_1\right)}{1000}$ and *λ*_2, max_(*λ*_1_), where *λ*_2, max_(*λ*_1_) was the lowest *λ*_2_ value for which FL returned a vector $\widehat{\beta}$ with all components equal. The optimal pair of tuning parameters (*λ*_1_, *λ*_2_) was selected as the one minimizing the prediction error estimated by 5-fold cross-validation [16], whose principle can be summarized as follows. The original sample is first partitioned into 5 equally sized subsamples. One subsample is held as the test set while the other 4 are used as a training set, on which FL estimates are computed for the 2500 values for (*λ*_1_, *λ*_2_). The prediction error is computed on the test set, and the process is repeated 5 times, and for each of the 2500 values of (*λ*_1_, *λ*_2_). The prediction error is defined as the averaged prediction error on the 5 test sets. FL analysis was implemented using the *FusedLasso* package.

Preprocessing steps and statistical analyses were carried out using the R software (https://www.r-project.org/) and the Bioconductor packages [21], including *lumi*, *wateRmelon*, and *sva* [29] for the preprocessing steps. The nominal level of statistical significance was set to 5%.

## Results

### Study population characteristics

Detailed characteristics of the 450 women included in the study are shown in Table 1. The average age at blood collection was 52 years (range 26–73). Participants had an average body mass index (BMI) of 26 kg/m^2^ (range 16–43) and were mostly post-menopausal (59%), never-smokers (56%), and moderately physically inactive (42%). The average daily intake of dietary folate was 270 μg/day (range 91–1012), and alcohol daily intake was 8 g/day (range 0–72). Non-alcohol consumers, defined as participants consuming less than 0.1 g/day of alcohol at recruitment, represented 15% of the population. Most participants were from the Italian and the German EPIC centers (Additional file 1: Figure S1).

**Table 1:** Characteristics of the study population (*n* = 450)

	Mean (SD)	Min-Max
Age at blood collection (years)	52 (9)	26–73
Weight (kg)	66 (11)	40–103
Height (cm)	161 (7)	143–196
BMI (kg/m^2^)	26 (4)	16–43
Alcohol intake (g/day)	8 (12)	0–72
Blood folate level (nmol/L)	15 (10)	1–89
Dietary folate (μg/day)	270 (106)	91–1012
Cd8t (%)	7.5 (4)	0–23
Cd4t (%)	13.5 (5)	0–34
Natural killer (%)	6.7 (5)	0–27
B cells (%)	6.1 (2)	0–17
Monocytes (%)	5.7 (3)	0–17
Granulocytes (%)	60.8 (9)	27–85
	*N*	%
Menopausal status		
Pre-menopause	186	41.3
Post-menopause	264	58.7
Smoking status		
Never	250	55.6
Former	93	20.7
Smoker	104	23.1
Missing	3	0.7
Physical activity index [58]		
Inactive	99	22.0
Moderate inactive	187	41.5
Moderate active	75	16.7
Active	78	10.7
Missing	11	2.4

*SD* standard deviation, reported for continuous variables only

### CpG site-specific models

After FDR correction, dietary folate intake was not significantly associated with methylation levels at any CpG sites (data not shown). Alcohol intake was inversely associated with the cg07382687 CpG site (*q*_val_ = 0.048) and positively associated with the cg03199996 site (*q*_val_ = 0.029) (Table 2). Both sites were located in an open sea region, i.e., a genomic region of isolated CpGs. cg07382687 was within the body region of gene *CREB3L2*, and cg03199996 was within the body region of gene *FAM65C*.

**Table 2:** CpG site-specific model results for the significant CpG sites for alcohol intake (adjusted for recruitment center, age at recruitment, menopausal status, and level of different lymphocyte subtypes)

CpG names		Alcohol intake			CpG characteristics			
		*β*_(1SD)_^1^	*q*_val_^2^	% change^3^	Associated genes	Gene region^4^	Island^5^	Chr
1	cg03199996	0.263	0.029	9.7	FAM65C	Body	Open sea	20
2	cg07382687	− 0.257	0.048	− 10.3	CREB3L2	Body	Open sea	7

^1^Coefficients for 1 standard deviation alcohol intake (SD = 11.8)

^2^False discovery rate (FDR) adjusted *p* values

^3^Percentage of methylation change for an increase of 1 SD increase of alcohol intake

^4^Gene region feature category describing the CpG position, from UCSC. *TSS200* 200 bases upstream of the transcriptional start site (TSS); *TSS1500* 1500 bases upstream of the TSS; *5′UTR* within the 5′ untranslated region, between the TSS and the ATG start site; *body* between the ATG and stop codon irrespective of the presence of introns, exons, TSS, or promoters; *3′UTR* between the stop codon and poly A signal

^5^The location of the CpG relative to the CpG island. *Shore* 0–2 kb from island, *Shelf* 2–4 kb from island, *N* upstream (5′) of CpG island, *S* downstream (3′) of CpG island, *open sea* isolated CpGs in the genome

### DMR analysis

**Fig. 2:**
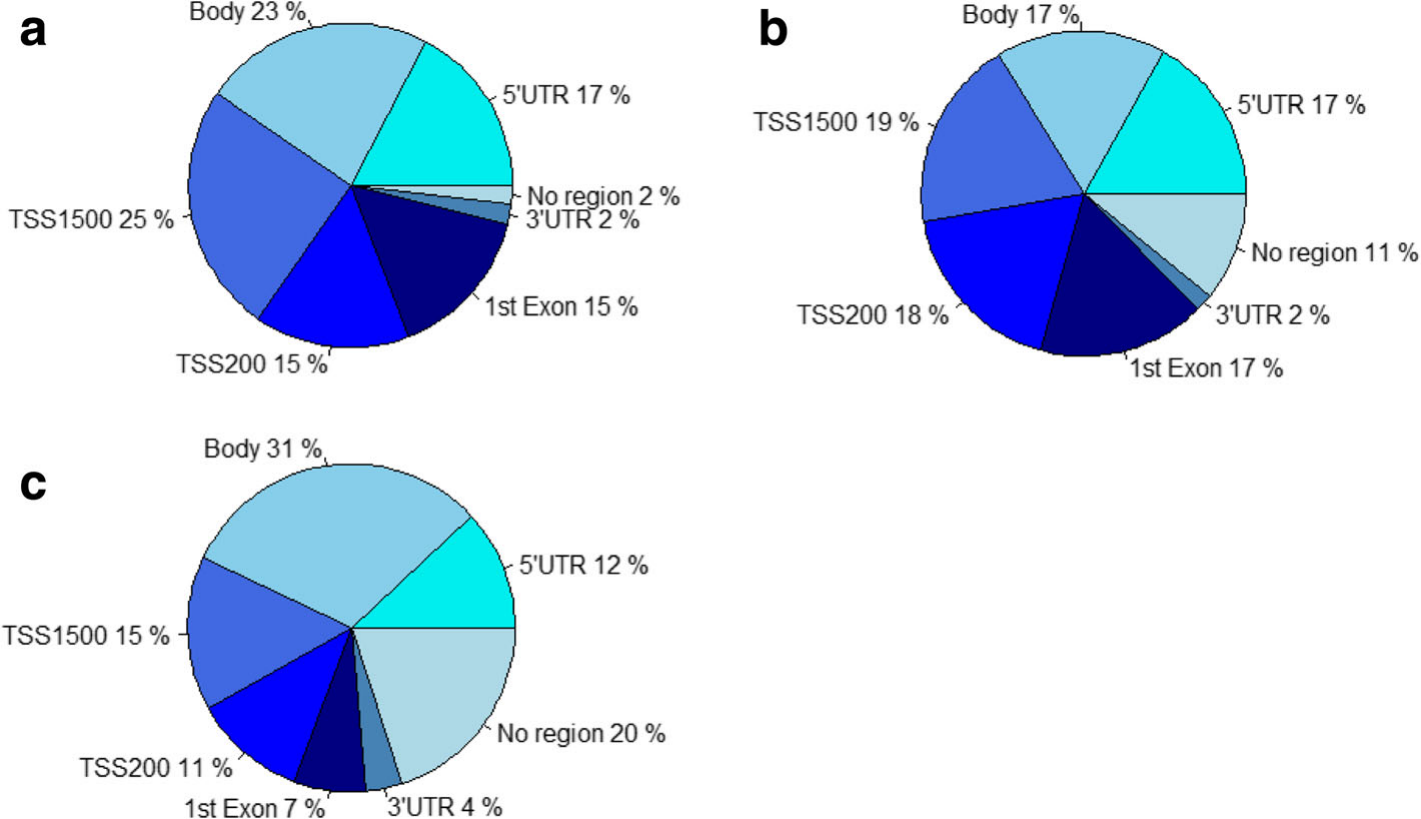
Repartition of gene regions (gene region feature category describing the CpG position, from UCSC. TSS200, 200 bases upstream of the transcriptional start site (TSS); TSS1500, 1500 bases upstream of the TSS; 5′UTR, within the 5′ untranslated region, between the TSS and the ATG start site; body, between the ATG and stop codon; irrespective of the presence of introns, exons, TSS, or promoters; 3′UTR, between the stop codon and poly A signal) among DMRs compare to their repartition within the Illumina 450K (the repartition of CpG sites was done among the 421,583 sites included in this study). **a** DMRs significant for folate. **b** DMRs significant for alcohol. **c** Illumina 450K

A total of 24 regions associated with dietary folate were identified, which included 190 CpG sites over-represented in the TSS1500 and 1st exon regions and under-represented in the body regions and regions outside any gene regions (Fig. 2a). The 15 most significant regions are described in Table 3 and the whole list provided in Additional file 2: Table S1. Among the 24 DMRs, 54% showed an inverse association with dietary folate, i.e., had a *β*_max, DMR_ < 0. The DMR most significantly associated with dietary folate (*q*_DMR_ = 1.3E−13, *β*_max, DMR_ = 0.019) was DMR.F1 in chromosome 7, including 49 CpG sites, related to *HOXA5* and *HOXA6* genes. DMR.F5 was associated with *HOXA4*, another gene of the homeobox family, (*q*_DMR_ = 5.8E−4, *β*_max, DMR_ = − 0.016).

**Table 3:** The 15 most significant DMRs associated with dietary folate out of 24 significant DMRs (adjusted for recruitment center, age at recruitment, menopausal status, and level of different lymphocyte subtypes)

	DMR characteristics				CpG characteristics			Fused lasso	
	Associated genes	Gene regions	hg19coord	Sites^1^	*q*_DMR_^2^	*β*_min, DMR_^3^	*β*_max, DMR_^3^	Overlap^4^	*β*_FL_^5^
F1	HOXA5,HOXA6	1stExon, 5′UTR, TSS200, TSS1500, 3′UTR, body	chr7:27183133-27185512	49	1.3E−13	0.0002	0.019		
F2	GDF7	Body	chr2:20869434-20871401	8	1.4E−08	− 0.016	− 0.033	7/8	− 0.0029
F3	CYP1A1	TSS1500	chr15:75018731-75019376	13	2.4E−05	0.0004	0.014		
F4	PRSS50	Body, 1stExon, 5′UTR, TSS200, TSS1500	chr3:46759096-46759698	9	2.4E−04	− 0.002	− 0.020	4/4	− 0.0069
F5	HOXA4	1stExon, 5′UTR, TSS200, TSS1500	chr7:27170241-27171154	14	5.8E−04	− 0.005	− 0.016		
F6	SYNGAP1	Body	chr6:33401192-33401542	6	1.0E−03	0.004	0.008		
F7	ZNF833	TSS1500, TSS200, body	chr19:11784514-11785337	13	1.1E−03	− 0.002	− 0.012		
F8	LAMB2	1stExon, 5′UTR, TSS200, TSS1500	chr3:49170496-49170849	6	3.1E−03	− 0.008	− 0.012		
F9	GPR19	5′UTR, 1stExon, TSS200, TSS1500	chr12:12848977-12849588	9	3.7E−03	0.001	0.023	6/7	0.0076
F10	MTMR15	TSS1500, TSS200, 5′UTR, 1stExon	chr15:31195612-31196075	7	4.0E−03	− 0.003	− 0.017		
F11	KCNE1	5′UTR, 1stExon, TSS200, TSS1500	chr21:35831871-35832364	8	4.2E−03	0.007	0.019		
F12	TNXB	Body	chr6:32054659-32055474	20	7.2E−03	0.0002	− 0.013		
F13	TERT	Body	chr5:1269992-1270152	3	7.2E−03	0.008	0.011		
F14	C2orf27A	5′UTR	chr2:132481613-132481826	2	1.7E−02	0.010	0.031		
F15	ANKRD44	Body	chr2:198029141-198029332	3	2.1E−02	− 0.005	− 0.018		

^1^Number of sites located in DMRs significant for dietary folate

^2^Minimum dietary folate *q* values of sites located in the DMRs (FDR correction)

^3^Absolute minimum and maximum of dietary folate coefficient of sites located in the DMRs, for 1 standard deviation of log-transformed diet folate (SD = 0.346)

^4^Number of sites from the FL region overlapping the DMR/number of sites in the FL region

^5^Dietary folate changes for an increase of 1 standard deviation of methylation levels of sites located in the FL region

Alcohol intake was associated with methylation levels in 90 DMRs, including 550 CpG sites over-represented in TSS200, 1st exon, and 5′ untranslated regions (5′UTR) and under-represented in the body regions and the regions outside any gene regions (Fig. 2b). The 15 most significant DMRs are detailed in Table 4, and the full list is described in Additional file 3: Table S2. Alcohol intake was positively associated with methylation levels in 66% of the 90 DMRs. The two sites associated with alcohol intake in the CpG site-specific analyses were not included in any DMRs. The most significant DMR associated with alcohol consumption was DMR.A1, 9 sites within the *GSDMD* gene, (*q*_DMR_ = 4.7E−14, *β*_max, DMR_ = 0.020).

**Table 4:** The 15 most significant DMRs associated with alcohol out of 90 significant DMRs (adjusted for recruitment center, age at recruitment, menopausal status, and level of different lymphocyte subtypes)

	DMRs characteristics				CpG characteristics			Fused lasso	
	Associated genes	Gene regions	hg19coord	Sites^1^	*q*_DMR_^2^	*β*_min, DMR_^3^	*β*_max, DMR_^3^	Overlap^4^	*β*_FL_^5^
A1	GSDMD	TSS1500, TSS200, 5′UTR, 1stExon	chr8:144635260-144636462	9	4.7E−14	0.0060	0.020		
A2			chr6:31650735-31651362	21	1.8E−13	0.0049	0.018	2/2, 2/2	0.390
A3	TRIM4	Body, 1stExon, 5′UTR, TSS200, TSS1500	chr7:99516603-99517509	14	3.0E−06	− 0.0007	0.018		
A4	RGL3	Body	chr19:11517079-11517436	5	3.3E−06	0.0041	0.020		
A5	COL9A3	TSS1500	chr20:61446962-61447992	32	4.8E−06	− 0.0004	− 0.012	4/4	− 1.027
A6	ADAM32	TSS1500, TSS200, 1stExon, 5′UTR, Body	chr8:38964500-38965492	10	1.3E−04	0.0019	0.014		
A7	C21orf56	5′UTR, 1stExon, TSS1500	chr21:47604052-47605174	8	1.5E−04	0.0191	0.032		
A8			chr2:118616155-118616576	5	1.9E−04	0.0143	0.019	5/7	0.514
A9	LTB4R2, LTB4R, CIDEB	Body, 1stExon, TSS1500, 5′UTR, TSS200	chr14:24780404-24780926	10	2.3E−04	− 0.0031	− 0.012	9/9	− 0.474
A10	PTDSS2	Body	chr11:457256-457304	3	3.0E−04	0.0044	0.011		
A11	SMC1B, RIBC2	Body, TSS1500, 1stExon, TSS200, 5′UTR	chr22:45808669-45810043	16	3.0E−04	0.0009	0.019	4/4, 3/3	0.332
A12			chr10:72013286-72013397	2	8.4E−04	− 0.0087	− 0.014		
A13	TRAF3	Body	chr14:103366987-103367858	5	1.4E−03	− 0.0044	0.013		
A14	C22orf27	TSS1500, TSS200, body	chr22:31317764-31318546	12	1.4E−03	0.0016	0.015	4/4, 2/2	0.641
A15	S100A13, S100A1	5′UTR, 1stExon, TSS1500, TSS200	chr1:153599479-153600156	8	3.0E−03	0.0076	0.019		

^1^Number of sites located in DMRs significant for alcohol

^2^Minimum alcohol *q* values of sites located in the DMRs (FDR correction)

^3^Absolute minimum and maximum of alcohol coefficient of sites located in the DMRs, for 1 standard deviation of alcohol intake (SD = 11.8)

^4^Number of sites from the FL region overlapping the DMR/number of sites in the FL region, appears twice if two FL regions are included in the DMR

^5^Alcohol changes for an increase of 1 standard deviation of methylation levels of sites located in the FL region or average of alcohol change if two FL regions are included in a DMR

Methylation levels of each CpG site located in the two most significant DMRs for folate and alcohol, i.e.DMR.F1, DMR.F2, DMR.A1 and DMR.A2, are presented in Additional file 4: Figure S2 by tertiles of dietary folate and alcohol intake, respectively. Correlation heatmaps of CpG sites in DMR.A1, DMR.A2, DMR.F1, and DMR.F2 are displayed in Additional file 5: Figure S3, showing high levels of correlation among methylation levels within the DMR.F2 of dietary folate and the DMR.A2 of alcohol. Other regions showed less correlation, including the DMR.A1 of alcohol intake.

### Fused lasso regression

For dietary folate, we identified 71 FL regions, 50 presenting a positive association and 21 an inverse association. Three FL regions were overlapping the 15 most significant DMRs (Table 3). Seven out of 8 sites from a FL region within the GDF7 gene were included in the DMR.F2 (*β*_FL_ = − 0.0029). All sites from a FL region associated with the *PRSS50* gene were part of the DMR.F4 (*β*_FL_ = − 0.0069). Six out of 7 sites from the FL region within the *GPR19* gene were within the DMR.F9 (*β*_FL_ = 0.0076). None of the 68 other FL regions were overlapping any folate-related DMRs.

For alcohol consumption, we identified 133 FL regions, 71 regions presenting a positive association and 62 an inverse association. Twenty-one regions were included in alcohol-related DMRs. Among them, 9 were overlapping 6 of the 15 most significant DMRs (Table 4). The situation where two close FL regions were part of the same DMR was observed 3 times in the 15 most significant alcohol-related DMRs. In particular, four and three sites from two FL regions located in chromosome 22 were included in DMR.A11, associated with genes *SMC1B* and *RIBC2*. All the 9 sites from a FL region were included in DMR.A9 (*β*_FL_ = − 0.474).

Graphical representations of the DMRs, the FL regions, and their overlap are illustrated for each chromosome in Additional file 6: Figure S4 for dietary folate and Additional file 7: Figure S5 for alcohol intake. For dietary folate, most of FL regions were located in chromosome 3, chromosome 22, and chromosome X. A maximum of four DMRs located in the same chromosome was observed for chromosomes 2 and 3. As for alcohol intake, DMR and FL showed overlap mostly in chromosomes 6 and 22, with, respectively, 4 and 3 DMRs overlapping FL regions.

## Discussion

In this study of women from a large prospective cohort, we investigated the association of dietary folate and alcohol intake with leukocyte DNA methylation via three different approaches. The site-specific analysis aimed at identifying single CpG sites independently from each other, whereas DMR and FL analyses aimed at identifying regions of CpG sites using the inter-correlation between methylation levels in close sites, thus exploiting the potential of specific regions of the epigenome to show methylation activity related to lifestyle factors.

While site-specific analysis showed a lack of association between dietary folate, alcohol intake, and individual CpG sites, DMR and FL analyses identified regions of the epigenome associated with dietary folate or alcohol intake. These two sites are located within the body region of the genes *FAMB65C* and *CREB3L2*. The *FAMB65C* gene, also named *RIPOR3*, is a non-annotated gene. The *CREB3L2* gene encodes a transcriptional activator protein and plays a critical role in cartilage development by activating the transcription of *SEC23A* [18]. Translocation of *CREB3L2* gene, located on chromosome 7, and the *FUS* gene (fused in sarcoma) located on the chromosome 16 has been found in some tumors, including skin cancer and soft tissue sarcoma [37, 38].

Alcohol is known to alter DNA methylation, mostly because it contributes to deregulation of folate absorption, which can lead to a dysfunction of OCM [27]. In our study, alcohol intake was associated with 90 DMRs, some of which may have a role in specific carcinogenesis processes. For example, alcohol intake was inversely associated with methylation levels in DMR.A64 related to the *MLH1* gene, which is frequently mutated in hereditary nonpolyposis colon cancer (HNPCC) [39]. A positive association between alcohol intake and methylation in the DMR.A79 was related to the *TSPAN32* (tetraspanin 32) gene, also known as the *TSSC6* gene, which is one of the several tumor suppressor genes located at locus 11p15.5 in the imprinted gene domain of chromosome 11 [28]. This locus has been associated with adrenocortical carcinoma, lung, ovarian, and breast cancers. Methylations within DMR.A1 were positively associated with alcohol intake, and the related *GSDMD* gene has also been suggested to act as a tumor suppressor [44]. Alcohol intake was also positively associated with DMR.A6 related to the gene *ADAM32*, which encodes a protein involved in diverse biological processes, such as brain development, fertilization, tumor development, and inflammation [36].

Several genes, associated with the 24 DMRs identified in our study for dietary folate, were possibly involved in biological processes leading to carcinogenesis. For example, dietary folate was positively associated with methylation in DMR.F16 related to the *RTKN* (rhotekin) gene, which interacts with GTP-bound Rho proteins. Rho proteins regulate many important cellular processes, including cell growth and transformation, cytokinesis, transcription, and smooth muscle contraction. Dysregulation of the Rho signal transduction pathway has been implicated in many forms of cancer such as bladder cancer, gastric cancer, and breast cancer [9, 15]. Dietary folate was also associated with methylation levels in DMR.F1 and DMR.F5 within the *HOXA4*, *HOXA5*, and *HOXA6* genes, members of the HOX family, known to be associated with cellular differentiation [46]. Perturbed HOX gene expression has been implicated in multiple cancer types [47]. In addition, *HOXA5* may also regulate gene expression and morphogenesis. Methylation of this gene may result in the loss of its expression and, since the encoded protein upregulates the tumor suppressor p53, may play an important role in tumorigenesis [55].

Results from site-specific and DMR analyses were generated with different analytical strategies: methylation levels in different sites were assumed independent in the former, with linear regression models fitted separately in each CpG site, while in the latter, the physical proximity of CpGs was exploited to identify specific regions of the epigenome with similar methylation activity, under the assumption that neighboring CpG sites may share relevant epigenetic information. FL analysis revealed some overlaps with DMRs, particularly for alcohol intake, where 9 FL regions were observed within the 15 most significant DMRs. Yet, the overlap between DMR and FL analyses is relatively low and their results deserve cautious interpretations as they have differences in analytical strategies. Unlike DMRs, FL does not take into account the physical distance between consecutive sites, but rather introduce smoothness of parameters estimated in adjacent mutually adjusted CpG sites. Methylation levels within a chromosome were mutually adjusted in FL regression, while in DMR analysis *t* test statistics were based on independent associations of methylation levels with folate and alcohol.

The association between folate and DNA methylation has been investigated at different stages of human life, in particular during fetal development and elderly, where folate is especially needed. A meta-analysis of mother-offspring pairs estimated the association between maternal plasma folate during pregnancy and DNA methylation in cord blood [25]. After FDR correction, maternal plasma folate was positively associated with methylation level at 27 CpG sites and inversely associated with methylation level at 416 CpG sites. None of these sites was observed in any of the 24 DMRs related to dietary folate in the present study. This might be explained by the lack of power to identify specific sites due to the sample size: over 2000 samples were included in Joubert’s meta-analysis against 450 in our study. Then, different methods were used to assess folate intake, i.e., plasma folate against dietary folate.

An intervention study was conducted to evaluate the effects of long-term supplementation with folic acid and vitamin B_12_ on white blood cell DNA methylation in elderly subjects [26]. After the intervention of 2 years, 162 sites were significantly differentially methylated compared to baseline, versus 6 sites only for the placebo group. Folate and vitamin B_12_ were not significantly associated with methylation level in any CpG sites. Within the same study, 173 and 425 DMRs were identified for folate and vitamin B_12_, respectively. The gene *HOX4*, which was inversely associated with dietary folate in our study in DMR.F5, was the only region overlapping with the first 10 DMRs found in the intervention study [26]. However, a higher level of folic acid was observed in the intervention study: averages blood folate of 52 and 23 nmol/L in the intervention and placebo groups, respectively, compared to an average blood folate of 15 nmol/L in our study which might partly explain the different findings.

Within a recent meta-analysis including 9643 participants of European ancestry, aged 42 to 76 years with 54% women [32], 363 CpG sites were significantly associated with alcohol consumption, with 87% of these sites showing inverse associations. In our study, site cg02711608 was part of the 363 identified sites and was also included in DMR.A25 associated with gene *SLC1A5*. *SLC1A5* gene encodes a protein which is a sodium-dependent amino acid transporter [42]. The important difference in the number of significant sites between the meta-analysis and the present study might mostly be explained by the larger study population size and the larger levels of alcohol intake observed in the meta-analysis [32]. Indeed, in the meta-analysis, composed of 46% of men, the medians of alcohol intake ranged from 0 to 14 g/day in the 10 European cohorts, while with a median of 3.5 g/day, alcohol intake was quite low in our study, which included only women. Lastly, cohort-specific approaches were used in the meta-analysis to remove technical variability, while the SVA approach was used in our study, which was shown to produce conservative findings compared to other normalizing techniques [40].

In our study, the sample size was relatively low (*n* = 450), and women only were included. With a median value of 3.5 g/day, a 95th percentiles equal to 31 g/day, and a percentage of non-consumers equal to 15%, alcohol intake displayed limited variability which potentially constrained the power of the study. In addition, questionnaire measurements used to assess dietary folate and alcohol intake are prone to exposure misclassification, which likely attenuated associations between lifestyle exposures and methylation levels. These elements may alone explain the lack of significant associations in our study. Further studies including men and women, possibly with larger sample size, are needed to further investigate the relationship between dietary folate, alcohol intake, and DNA methylation.

A major strength of this study was the use of ad hoc methodology for normalization of methylation data. Technical management of samples likely introduces systematic technical variability in methylation measurements that might compromise the accuracy of the acquisition process and, if not properly taken into account, could introduce bias in the estimation of the association of interest. The population used in this study included European women from the UK, Germany, Italy, Greece, The Netherlands, and Spain, implying a diversity of diet and lifestyle habits. Three approaches were used to evaluate the relationship between dietary folate, alcohol intake, and DNA methylation. The comparison between DMR and FL analyses was particularly relevant to identify regions of the genome associated with dietary folate and alcohol intake.

Alcohol was classified as group 1 carcinogen in 2012 by the IARC Monograph [22] and was associated with cancer of the upper aero-digestive tract, female breast, liver, and colorectum. Dietary folate has been recently inversely associated with the risk of breast cancer in EPIC [12], although the evidence is not conclusive [59]. Among the DMRs identified in this study for dietary folate or alcohol intake, several regions were associated with genes potentially implicated in cancer development, such as *RTKN*, the *HOX* family of genes, and the two tumor suppressor genes *GSDMD* and *TSPAN32*. Our study provides some evidence that dietary folate and alcohol intakes may be associated with carcinogenesis through a deregulation of epigenetic mechanisms, although our findings need to be replicated in future evaluations.

In this study, site-specific analyses served as a basis to explore more complex evaluations. By addressing the high dimensionality and complexity of DNA methylation, statistical techniques used in this work may prove useful for future epigenetic studies focusing on the relationship between lifestyle exposures, DNA methylation, and the occurrence of disease outcomes. These tools presented may be adapted to suit specific features of other *-omics* data.

## Conclusion

Weak associations between alcohol intake and methylation levels at two CpG sites were observed. DMR and FL analyses provided evidence that specific regions of CpG sites were associated with dietary folate and alcohol intake, assuming that neighboring features share relevant epigenetic information. Folate and alcohol are known not only to be associated with breast cancer but also to have a mutually antagonistic role in the one-carbon metabolism. In some regions identified by DMRs or FL analysis, mapped genes are known to act as tumor suppressors such as the *GSDMD* and *HOXA5* genes. These results were in line with the hypothesis that folate- and alcohol-deregulated epigenetic mechanisms might have a role in the pathogenesis of cancer.

## Additional files

Additional file 1: **Figure S1.** Sample size by recruitment centers. (PDF 10 kb)

Additional file 2: **Table S1.** DMRs associated with dietary folate (log). (DOCX 19 kb)

Additional file 3: **Table S2.** DMRs associated with alcohol intake. (DOCX 34 kb)

Additional file 4: **Figure S2.** Graphical representation of the most 2 significant DMR of dietary folate and alcohol intake. The *x*-axis represents the position (hg 19 coordinates) of the CpGs included in the plotted DMR. Each tertile of dietary folate, alcohol intake, or their interaction is represented by different colors: green for T1, blue for T2, and red for T3. For all the CpGs included in the plotted DMR, the dashed lines are their 1st and 3rd quartiles of methylation levels and the points represent their median values. (PDF 33 kb)

Additional file 5: **Figure S3.** Correlation heatmap of methylation levels inside the two most significant DMR of folate and alcohol. (PDF 43 kb)

Additional file 6: **Figure S4.** DMRs and FL regions of folate in each chromosome. Dark blue rectangles represent DMRs and light blue FL regions. Overlaps between the two methods are represented by red points. Positive coefficients of the two methods are represented on the top part of each graphic, and negative coefficients are on the bottom part. Positive (negative) coefficients of DMRs were set to 0.5 (− 0.5) and positive (negative) coefficients of FL regions were set to 1 (− 1) to clearly differentiate DMRs from FL regions. The *x*-axis represents the rank of CpG sites according to their position on the chromosome. (PDF 12 kb)

Additional file 7: **Figure S5.** DMRs and FL regions of alcohol in each chromosome. Dark blue rectangles represent DMRs and light blue FL regions. Overlaps between the two methods are represented by red points. Positive coefficients of the two methods are represented on the top part of each graphic and negative coefficients are on the bottom part. Positive (negative) coefficients of DMRs were set to 0.5 (− 0.5), and positive (negative) coefficients of FL regions were set to 1 (− 1) to clearly differentiate DMRs from FL regions. The *x*-axis represents the rank of CpG sites according to their position on the chromosome. (PDF 58 kb)

## Abbreviations

BC: Breast cancer
BMI: Body mass index
DMR: Differentially methylated region
EPIC: European Prospective Investigation into Cancer and Nutrition
FDR: False discovery rate
FL: Fused lasso
HM450K: Illumina Infinium HumanMethylation 450K
MAF: Minor allele frequency
NK: Natural killer
OCM: One-carbon metabolite
SAM: *S*-Adenosylmethionine
SVA: Surrogate variable analysis
