# Supplementary figures and images for: Association of leukocyte DNA methylation changes with dietary folate and alcohol intake in the EPIC study
Source: Clin Epigenetics. 2019 Apr 2;11:57. doi: 10.1186/s13148-019-0637-x (PMC6444439; doi:10.1186/s13148-019-0637-x)

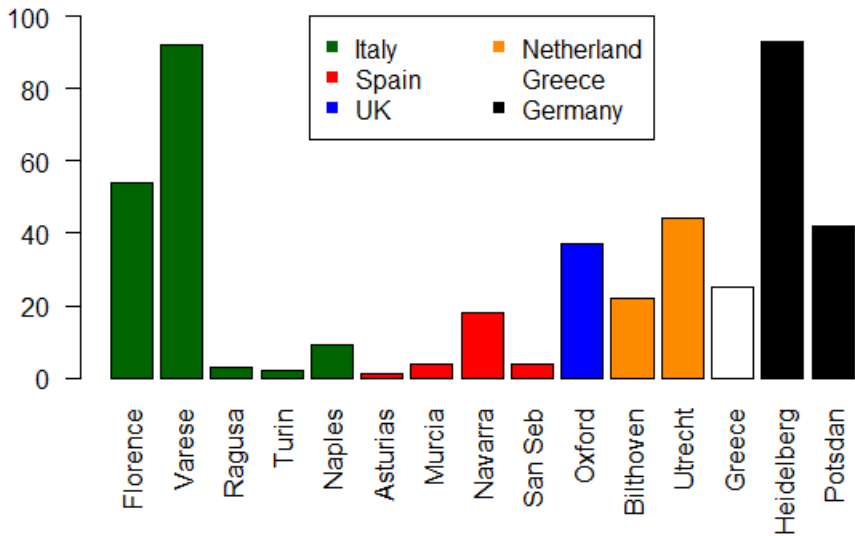

Supplement: Supplementary file 1 — Figure S1. Sample size by recruitment centers. (PDF 10 kb) [file 13148_2019_637_MOESM1_ESM.pdf]

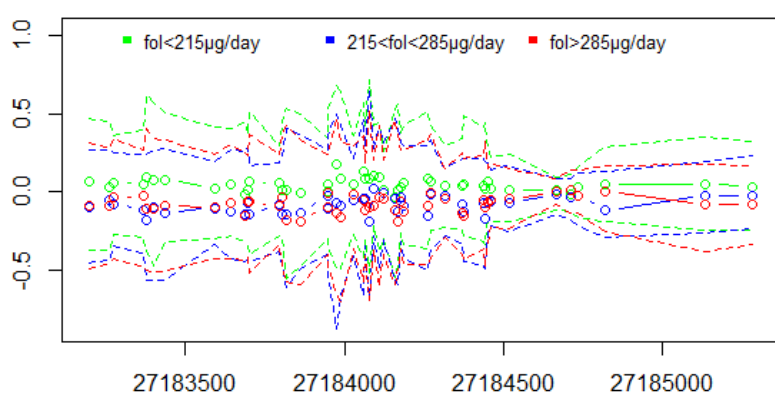

A) Dietary folate: DMR.F1

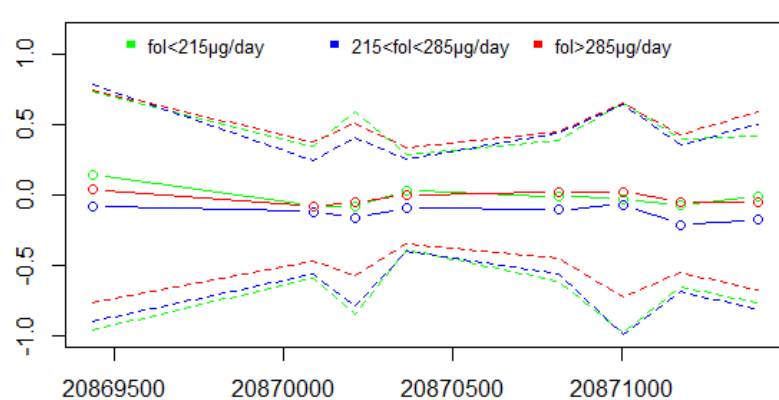

B) Dietary folate: DMR.F2

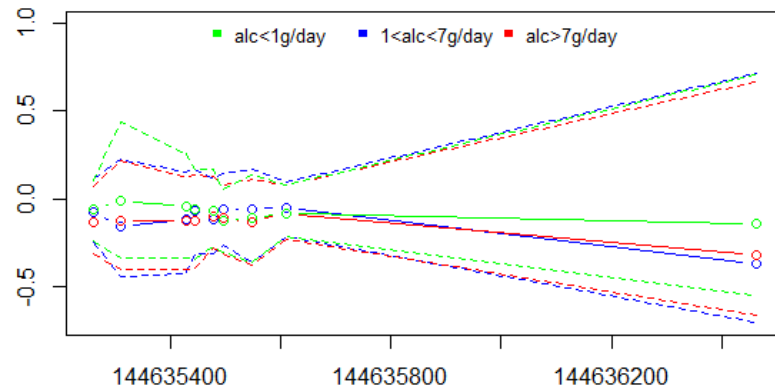

C) Alcohol intake: DMR.A1

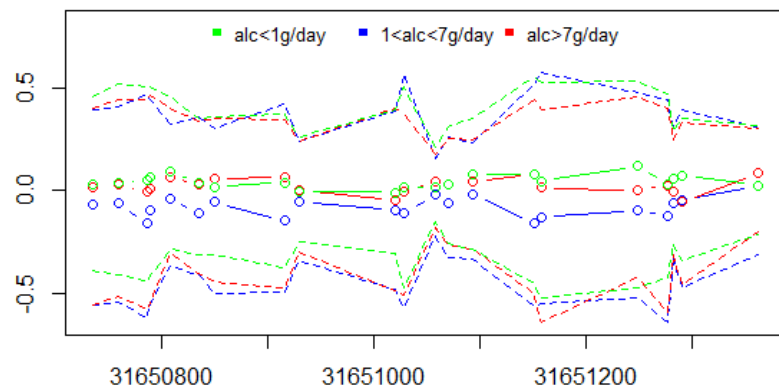

D) Alcohol intake: DMR.A2

Supplement: Supplementary file 4 — Figure S2. Graphical representation of the most 2 significant DMR of dietary folate and alcohol intake. The x-axis represents the position (hg 19 coordinates) of the CpGs included in the plotted DMR. Each tertile of dietary folate, alcohol intake, or their interaction is represented by different colors: green for T1, blue for T2, and red for T3. For all the CpGs included in the plotted DMR, the dashed lines are their 1st and 3rd quartiles of methylation levels and the points represent their median values. (PDF 33 kb) [file 13148_2019_637_MOESM4_ESM.pdf]

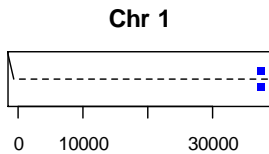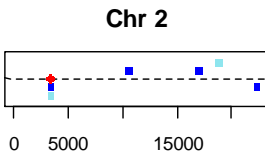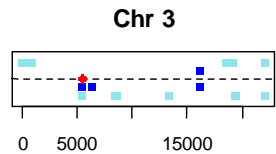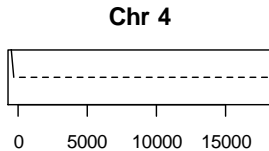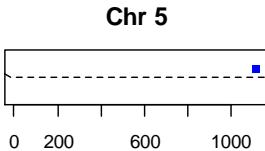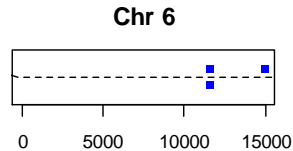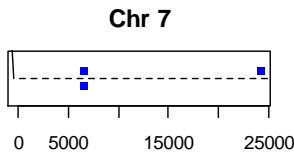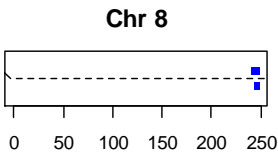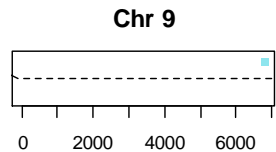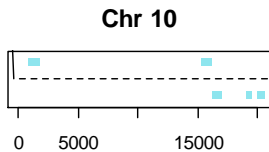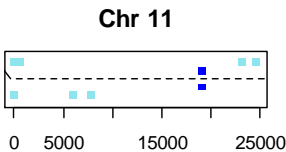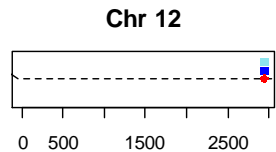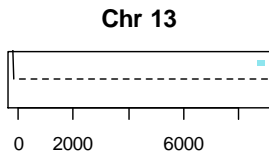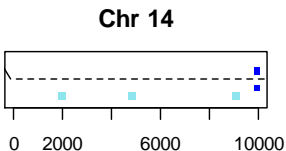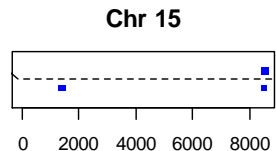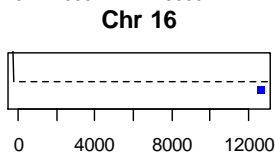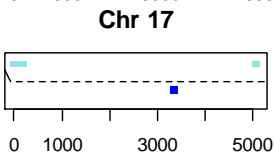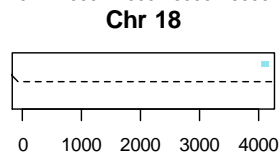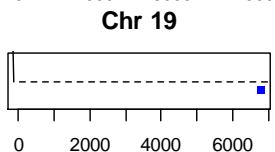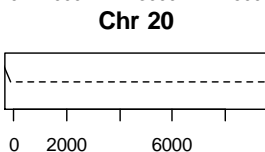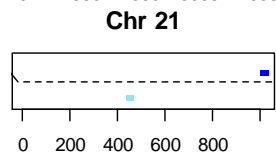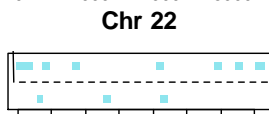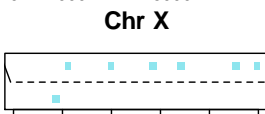

Supplement: Supplementary file 6 — Figure S4. DMRs and FL regions of folate in each chromosome. Dark blue rectangles represent DMRs and light blue FL regions. Overlaps between the two methods are represented by red points. Positive coefficients of the two methods are represented on the top part of each graphic, and negative coefficients are on the bottom part. Positive (negative) coefficients of DMRs were set to 0.5 (− 0.5) and positive (negative) coefficients of FL regions were set to 1 (− 1) to clearly differentiate DMRs from FL regions. The x-axis represents the rank of CpG sites according to their position on the chromosome. (PDF 12 kb) [file 13148_2019_637_MOESM6_ESM.pdf]

Chr 1

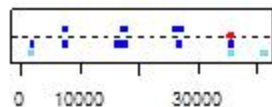

Chr 2

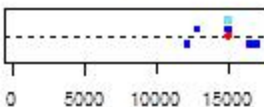

Chr 3

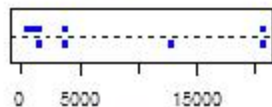

Chr 4

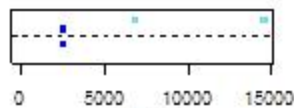

Chr 5

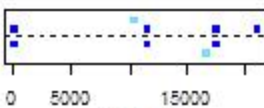

Chr 6

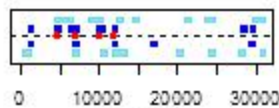

Chr 7

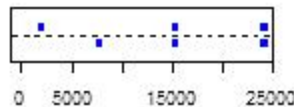

Chr 8

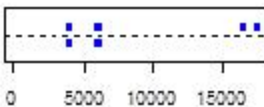

Chr 9

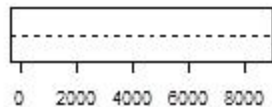

Chr 10

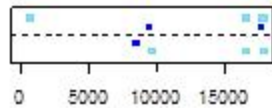

**Chr 11**

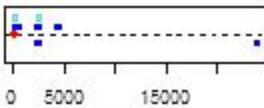

Chr 12

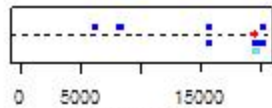

Chr 13

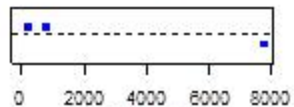

Chr 14

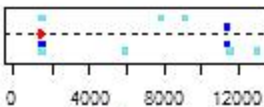

Chr 15

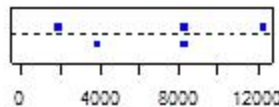

Chr 16

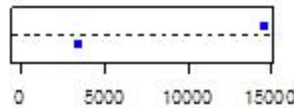

Chr 17

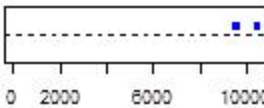

Chr 18

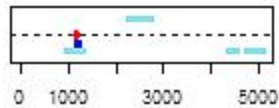

Chr 19

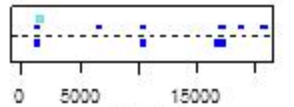

Chr 20

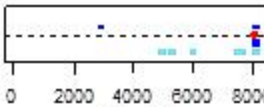

Chr 21

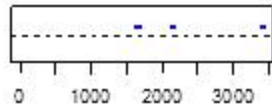

Chr 22

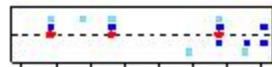

Chr X

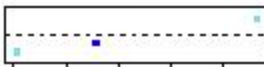

Supplement: Supplementary file 7 — Figure S5. DMRs and FL regions of alcohol in each chromosome. Dark blue rectangles represent DMRs and light blue FL regions. Overlaps between the two methods are represented by red points. Positive coefficients of the two methods are represented on the top part of each graphic and negative coefficients are on the bottom part. Positive (negative) coefficients of DMRs were set to 0.5 (− 0.5), and positive (negative) coefficients of FL regions were set to 1 (− 1) to clearly differentiate DMRs from FL regions. The x-axis represents the rank of CpG sites according to their position on the chromosome. (PDF 58 kb) [file 13148_2019_637_MOESM7_ESM.pdf]
