# Supplementary material for: Association of leukocyte DNA methylation changes with dietary folate and alcohol intake in the EPIC study
Source: Clin Epigenetics. 2019 Apr 2;11:57. doi: 10.1186/s13148-019-0637-x (PMC6444439; doi:10.1186/s13148-019-0637-x)
Supplement: Supplementary file 2 — Table S1. DMRs associated with dietary folate (log). (DOCX 19 kb) [file 13148_2019_637_MOESM2_ESM.docx]

|  | **Associated genes** | **Gene regions** | **hg19coord** | **Sites^2^** | $\boldsymbol{q}_{\boldsymbol{DMR}}$**^3^** | $\boldsymbol{\beta}_{\boldsymbol{min,DMR}}$**^4^** | $\boldsymbol{\beta}_{\boldsymbol{max,DMR}}$**^4^** |
| --- | --- | --- | --- | --- | --- | --- | --- |
| 1 | HOXA5,HOXA6 | 1stExon,5'UTR,TSS200,TSS1500,3'UTR,Body | chr7:27183133-27185512 | 49 | 1.3E-13 | 0.0002 | 0.019 |
| 2 | GDF7 | Body | chr2:20869434-20871401 | 8 | 1.4E-08 | -0.016 | -0.033 |
| 3 | CYP1A1 | TSS1500 | chr15:75018731-75019376 | 13 | 2.4E-05 | 0.0004 | 0.014 |
| 4 | PRSS50 | Body,1stExon,5'UTR,TSS200,TSS1500 | chr3:46759096-46759698 | 9 | 2.4E-04 | -0.002 | -0.020 |
| 5 | HOXA4 | 1stExon,5'UTR,TSS200,TSS1500 | chr7:27170241-27171154 | 14 | 5.8E-04 | -0.005 | -0.016 |
| 6 | SYNGAP1 | Body | chr6:33401192-33401542 | 6 | 1.0E-03 | 0.004 | 0.008 |
| 7 | ZNF833 | TSS1500,TSS200,Body | chr19:11784514-11785337 | 13 | 1.1E-03 | -0.002 | -0.012 |
| 8 | LAMB2 | 1stExon,5'UTR,TSS200,TSS1500 | chr3:49170496-49170849 | 6 | 3.1E-03 | -0.008 | -0.012 |
| 9 | GPR19 | 5'UTR,1stExon,TSS200,TSS1500 | chr12:12848977-12849588 | 9 | 3.7E-03 | 0.001 | 0.023 |
| 10 | MTMR15 | TSS1500,TSS200,5'UTR,1stExon | chr15:31195612-31196075 | 7 | 4.0E-03 | -0.003 | -0.017 |
| 11 | KCNE1 | 5'UTR,1stExon,TSS200,TSS1500 | chr21:35831871-35832364 | 8 | 4.2E-03 | 0.007 | 0.019 |
| 12 | TNXB | Body | chr6:32054659-32055474 | 20 | 7.2E-03 | 0.0002 | -0.013 |
| 13 | TERT | Body | chr5:1269992-1270152 | 3 | 7.2E-03 | 0.008 | 0.011 |
| 14 | C2orf27A | 5'UTR | chr2:132481613-132481826 | 2 | 1.7E-02 | 0.010 | 0.031 |
| 15 | ANKRD44 | Body | chr2:198029141-198029332 | 3 | 2.1E-02 | -0.005 | -0.018 |
| 16 | RTKN | Body,TSS1500 | chr2:74668072-74668286 | 2 | 2.9E-02 | 0.006 | 0.008 |
| 17 | PTPRN2 | Body | chr7:157406607-157406737 | 2 | 3.0E-02 | 0.011 | 0.012 |
| 18 | PANX1 | Body | chr11:93862716-93862749 | 2 | 3.5E-02 | -0.009 | 0.009 |
| 19 | CDH3 | 1stExon,5'UTR | chr16:68678841-68678998 | 2 | 3.7E-02 | -0.002 | -0.008 |
| 20 |  |  | chr8:713162-713216 | 3 | 3.8E-02 | 0.002 | 0.012 |
| 21 | LEFTY2 | TSS1500 | chr1:226129481-226129561 | 2 | 3.9E-02 | 0.001 | -0.015 |
| 22 | EML1 | TSS1500 | chr14:100259329-100259352 | 3 | 4.1E-02 | 0.001 | -0.013 |
| 23 | CHRNB1 | Body | chr17:7350244-7350282 | 2 | 4.1E-02 | -0.008 | -0.010 |
| 24 | PAQR9 | TSS1500 | chr3:142682652-142682682 | 2 | 4.2E-02 | 0.005 | -0.009 |

^1^ Adjusted for alcohol, recruitment centre, age at recruitment, menopausal status, level of different lymphocyte subtypes and BC status;

^2^ Number of sites located in DMRs significant for dietary folate;

^3^ Minimum dietary folate q-values of sites located in the DMRs (FDR correction);

^4^ Absolute minimum and maximum of dietary folate coefficients of sites located in the DMRs, for 1 standard deviation of log-transformed diet folate (SD=0.346).
