# Supplementary material for: Association of leukocyte DNA methylation changes with dietary folate and alcohol intake in the EPIC study
Source: Clin Epigenetics. 2019 Apr 2;11:57. doi: 10.1186/s13148-019-0637-x (PMC6444439; doi:10.1186/s13148-019-0637-x)
Supplement: Supplementary file 3 — Table S2. DMRs associated with alcohol intake. (DOCX 34 kb) [file 13148_2019_637_MOESM3_ESM.docx]

|  | **Associated genes** | **Gene regions** | **hg19coord** | **Sites^2^** | $\boldsymbol{q}_{\boldsymbol{DMR}}$**^3^** | $\boldsymbol{\beta}_{\boldsymbol{min,DMR}}$**^4^** | $\boldsymbol{\beta}_{\boldsymbol{max,DMR}}$**^4^** |
| --- | --- | --- | --- | --- | --- | --- | --- |
| 1 | GSDMD | TSS1500, TSS200, 5'UTR, 1stExon | chr8:144635260-144636462 | 9 | 4,7E-14 | 0.0060 | 0.020 |
| 2 |  |  | chr6:31650735-31651362 | 21 | 1,8E-13 | 0.0049 | 0.018 |
| 3 | TRIM4 | Body, 1stExon, 5'UTR, TSS200, TSS1500 | chr7:99516603-99517509 | 14 | 3,0E-06 | -0.0007 | 0.018 |
| 4 | RGL3 | Body | chr19:11517079-11517436 | 5 | 3,3E-06 | 0.0041 | 0.020 |
| 5 | COL9A3 | TSS1500 | chr20:61446962-61447992 | 32 | 4,8E-06 | -0.0004 | -0.012 |
| 6 | ADAM32 | TSS1500, TSS200, 1stExon, 5'UTR, Body | chr8:38964500-38965492 | 10 | 1,3E-04 | 0.0019 | 0.014 |
| 7 | C21orf56 | 5'UTR, 1stExon, TSS1500 | chr21:47604052-47605174 | 8 | 1,5E-04 | 0.0191 | 0.032 |
| 8 |  |  | chr2:118616155-118616576 | 5 | 1,9E-04 | 0.0143 | 0.019 |
| 9 | LTB4R2, LTB4R, CIDEB | Body, 1stExon, TSS1500, 5'UTR, TSS200 | chr14:24780404-24780926 | 10 | 2,3E-04 | -0.0031 | -0.012 |
| 10 | PTDSS2 | Body | chr11:457256-457304 | 3 | 3,0E-04 | 0.0044 | 0.011 |
| 11 | SMC1B, RIBC2 | Body, TSS1500, 1stExon, TSS200, 5'UTR | chr22:45808669-45810043 | 16 | 3,0E-04 | 0.0009 | 0.019 |
| 12 |  |  | chr10:72013286-72013397 | 2 | 8,4E-04 | -0.0087 | -0.014 |
| 13 | TRAF3 | Body | chr14:103366987-103367858 | 5 | 1,4E-03 | -0.0044 | 0.013 |
| 14 | C22orf27 | TSS1500, TSS200, Body | chr22:31317764-31318546 | 12 | 1,4E-03 | 0.0016 | 0.015 |
| 15 | S100A13, S100A1 | 5'UTR, 1stExon, TSS1500, TSS200 | chr1:153599479-153600156 | 8 | 3,0E-03 | 0.0076 | 0.019 |
| 16 | VARS | Body | chr6:31760521-31761076 | 12 | 3,1E-03 | 0.0012 | 0.007 |
| 17 |  |  | chr13:20781097-20781165 | 3 | 3,2E-03 | 0.0071 | 0.011 |
| 18 |  |  | chr6:5783800-5783863 | 2 | 3,3E-03 | -0.0038 | 0.016 |
| 19 | TSSK6, NDUFA13 | 1stExon, TSS1500, 5'UTR, TSS200 | chr19:19625761-19626599 | 6 | 3,8E-03 | 0.0034 | 0.010 |
| 20 |  |  | chr6:27637302-27637537 | 4 | 3,9E-03 | 0.0056 | 0.014 |
| 21 | THUMPD3 | TSS1500, TSS200, 5'UTR, 1stExon | chr3:9404422-9405070 | 9 | 4,5E-03 | 0.0045 | 0.013 |
| 22 | NKX2-6 | Body, TSS200, TSS1500 | chr8:23562918-23564294 | 12 | 4,9E-03 | 0.0006 | 0.011 |
| 23 | ATP2B2 | 3'UTR, Body | chr3:10370264-10370704 | 4 | 5,5E-03 | 0.0069 | 0.010 |
| 24 |  |  | chr6:30094947-30095802 | 26 | 5,5E-03 | -0.0014 | -0.011 |
| 25 | SLC1A5 | Body, 5'UTR, 1stExon, TSS200 | chr19:47287778-47288263 | 6 | 5,5E-03 | -0.0024 | -0.008 |
| 26 | PCSK4 | Body | chr19:1486986-1487605 | 5 | 5,7E-03 | 0.0018 | 0.014 |
| 27 | IRF6 | 5'UTR, 1stExon, TSS200, TSS1500 | chr1:209979111-209979779 | 8 | 5,7E-03 | -0.0044 | -0.021 |
| 28 | C7orf16 | TSS1500, TSS200, 5'UTR, 1stExon | chr7:31726494-31726912 | 6 | 6,1E-03 | -0.0038 | -0.014 |
| 29 | ADM2, MIOX | 3'UTR, TSS1500, TSS200, 5'UTR, 1stExon | chr22:50924745-50925337 | 5 | 6,3E-03 | 0.0015 | 0.015 |
| 30 | DUPD1 | Body | chr10:76803669-76803925 | 3 | 6,4E-03 | 0.0043 | 0.009 |
| 31 | GOLPH3L | TSS1500 | chr1:150670196-150670422 | 2 | 6,9E-03 | 0.0078 | 0.023 |
| 32 | FLJ44606 | 5'UTR, TSS200, TSS1500 | chr5:126408756-126409573 | 13 | 7,4E-03 | 0.0012 | 0.017 |
| 33 | C2orf27B | 5'UTR, 1stExon, TSS200, TSS1500 | chr2:132558939-132559484 | 6 | 7,4E-03 | -0.0006 | -0.015 |
| 34 |  |  | chr7:157294107-157294502 | 5 | 7,6E-03 | -0.0007 | 0.014 |
| 35 | ALDH7A1 | 1stExon, TSS200 | chr5:125930870-125931275 | 6 | 7,9E-03 | 0.0035 | 0.012 |
| 36 | HGFAC | Body | chr4:3449663-3449904 | 3 | 8,6E-03 | -0.0022 | 0.013 |
| 37 |  |  | chr2:129494526-129494877 | 4 | 8,7E-03 | -0.0007 | -0.007 |
| 38 | GABBR1 | Body | chr6:29599012-29599390 | 10 | 8,9E-03 | 0.0023 | 0.008 |
| 39 | SLC39A4 | Body | chr8:145638202-145639181 | 5 | 8,9E-03 | 0.0003 | 0.015 |
| 40 |  |  | chr11:8361190-8361530 | 3 | 8,9E-03 | 0.0143 | 0.016 |
| 41 | C17orf98 | 1stExon, TSS200 | chr17:36997449-36997740 | 5 | 8,9E-03 | 0.0103 | 0.015 |
| 42 |  |  | chr11:128557481-128557965 | 3 | 9,6E-03 | -0.0039 | -0.010 |
| 43 | ZAP70 | 5'UTR, Body | chr2:98340425-98340921 | 4 | 1,1E-02 | 0.0041 | 0.017 |
| 44 | SFRS8 | Body | chr12:132270218-132270829 | 4 | 1,2E-02 | 0.0004 | -0.014 |
| 45 | LOC654342 | TSS200, TSS1500 | chr2:91847976-91848218 | 8 | 1,2E-02 | -0.0004 | -0.009 |
| 46 | SNORD46, RPS8, SNORD38A | TSS200, Body, TSS1500 | chr1:45242073-45242356 | 3 | 1,3E-02 | -0.0056 | -0.009 |
| 47 |  |  | chr5:80493-80900 | 2 | 1,3E-02 | -0.0068 | 0.024 |
| 48 |  |  | chr22:50165244-50165512 | 3 | 1,4E-02 | -0.0066 | -0.016 |
| 49 | C21orf88 | TSS1500 | chr21:40985387-40985406 | 2 | 1,5E-02 | 0.0009 | 0.011 |
| 50 | PPT2, PRRT1 | TSS1500, TSS200 | chr6:32120623-32121261 | 23 | 1,5E-02 | 0.0011 | -0.012 |
| 51 |  |  | chr6:167559913-167560727 | 4 | 1,6E-02 | -0.0107 | -0.017 |
| 52 | ADAM12 | 1stExon, 5'UTR | chr10:128076910-128076941 | 3 | 1,6E-02 | 0.0042 | 0.010 |
| 53 |  |  | chr13:110521956-110522297 | 5 | 1,7E-02 | -0.0055 | -0.020 |
| 54 | GABRG2 | TSS1500 | chr5:161494015-161494307 | 3 | 1,7E-02 | -0.0036 | 0.017 |
| 55 | ATHL1 | 5'UTR, Body | chr11:289774-290292 | 4 | 1,7E-02 | 0.0073 | 0.029 |
| 56 | SHANK1 | Body | chr19:51171061-51171247 | 2 | 1,7E-02 | 0.0078 | 0.014 |
| 57 | SST | 1stExon, 5'UTR, TSS200 | chr3:187388128-187388281 | 4 | 1,7E-02 | 0.0005 | 0.010 |
| 58 | IQSEC1 | Body, 5'UTR, 1stExon, TSS200 | chr3:13114343-13114803 | 6 | 1,8E-02 | 0.0008 | -0.013 |
| 59 |  |  | chr15:96911165-96911531 | 2 | 1,8E-02 | 0.0063 | 0.007 |
| 60 | ANKRD30B | TSS1500, TSS200, 1stExon, 5'UTR | chr18:14747888-14748298 | 9 | 1,9E-02 | -0.0022 | -0.013 |
| 61 | OTUD5 | 5'UTR, 1stExon, TSS200, TSS1500 | chrX:48814580-48815125 | 9 | 2,0E-02 | -0.0003 | -0.006 |
| 62 | SDR42E1 | 5'UTR, 1stExon, TSS200 | chr16:82044738-82045183 | 7 | 2,1E-02 | 0.0053 | 0.012 |
| 63 | ISLR2 | Body, 3'UTR | chr15:74427234-74427499 | 3 | 2,3E-02 | -0.0073 | 0.010 |
| 64 | EPM2AIP1, MLH1 | 1stExon, TSS1500 | chr3:37033625-37033903 | 5 | 2,4E-02 | 0.0017 | -0.013 |
| 65 |  |  | chr21:44253516-44253611 | 2 | 2,5E-02 | 0.0069 | 0.008 |
| 66 | TNFRSF19 | TSS1500, TSS200, 1stExon, 5'UTR | chr13:24144483-24144985 | 6 | 2,5E-02 | 0.0017 | 0.011 |
| 67 |  |  | chr5:180541684-180541897 | 3 | 2,6E-02 | 0.0053 | 0.012 |
| 68 | CCDC42B | Body | chr12:113592319-113592619 | 4 | 3,0E-02 | -0.0017 | -0.008 |
| 69 | SCARF2 | Body | chr22:20783497-20783963 | 3 | 3,1E-02 | 0.0077 | 0.015 |
| 70 | SHISA7 | 1stExon | chr19:55953951-55953996 | 2 | 3,2E-02 | 0.0040 | 0.016 |
| 71 | LOC285796 | TSS1500 | chr6:163746113-163746175 | 2 | 3,3E-02 | 0.0084 | 0.012 |
| 72 | COX4I2 | TSS200, 1stExon, 5'UTR | chr20:30225517-30225851 | 6 | 3,3E-02 | 0.0043 | 0.009 |
| 73 | CLDN9 | 1stExon, 5'UTR | chr16:3062597-3062975 | 4 | 3,4E-02 | -0.0044 | -0.008 |
| 74 | MAD1L1 | Body | chr7:1896154-1896220 | 2 | 3,4E-02 | 0.0087 | 0.012 |
| 75 | PARP9, DTX3L | 5'UTR, TSS1500 | chr3:122281881-122281975 | 3 | 3,5E-02 | -0.0029 | -0.011 |
| 76 | PRKCZ | 5'UTR, Body | chr1:2058417-2058790 | 2 | 3,5E-02 | -0.0121 | -0.016 |
| 77 | HOXC8 | TSS1500, TSS200 | chr12:54402431-54402717 | 7 | 3,6E-02 | 0.0039 | 0.018 |
| 78 | ACTC1 | Body | chr15:35086890-35086985 | 4 | 3,6E-02 | 0.0028 | 0.012 |
| 79 | C11orf21, TSPAN32 | Body, TSS1500, 1stExon, TSS200 | chr11:2322500-2323083 | 13 | 3,7E-02 | 0.0011 | 0.010 |
| 80 | C6orf123 | TSS200, TSS1500 | chr6:168197699-168197983 | 4 | 3,9E-02 | 0.0011 | -0.014 |
| 81 |  |  | chr6:41376604-41376993 | 3 | 4,0E-02 | -0.0049 | -0.014 |
| 82 | KRT26 | 1stExon, 5'UTR | chr17:38928324-38928380 | 2 | 4,1E-02 | 0.0015 | 0.022 |
| 83 | ASCL2 | TSS1500 | chr11:2292890-2293117 | 8 | 4,3E-02 | 0.0008 | -0.011 |
| 84 | LCE3A | 1stExon | chr1:152595322-152595351 | 2 | 4,4E-02 | -0.0111 | -0.012 |
| 85 | PIWIL1 | TSS200, 1stExon, 5'UTR | chr12:130822603-130822674 | 3 | 4,5E-02 | -0.0131 | -0.025 |
| 86 | C1orf163 | Body | chr1:53163523-53163758 | 3 | 4,5E-02 | -0.0030 | 0.008 |
| 87 |  |  | chr19:48675305-48676053 | 2 | 4,7E-02 | -0.0022 | 0.006 |
| 88 | TMEM51 | 5'UTR | chr1:15541182-15541349 | 4 | 4,8E-02 | -0.0015 | 0.008 |
| 89 | FKBP11 | TSS200, TSS1500 | chr12:49319500-49319673 | 3 | 4,9E-02 | 0.0022 | 0.007 |
| 90 | TRIM69 | TSS1500 | chr15:45028083-45028098 | 2 | 5,0E-02 | -0.0034 | -0.004 |

^1^ Adjusted for recruitment centre, age at recruitment, menopausal status, level of different lymphocyte subtypes and BC status;

^2^ Number of sites located in DMRs significant for alcohol;

^3^ Minimum alcohol q-values of sites located in the DMRs (FDR correction);

^4^ Absolute minimum and maximum of alcohol coefficients of sites located in the DMRs, for 1 standard deviation of alcohol intake (SD=11.8).
