# Supplementary material for: Association of leukocyte DNA methylation changes with dietary folate and alcohol intake in the EPIC study
Source: Clin Epigenetics. 2019 Apr 2;11:57. doi: 10.1186/s13148-019-0637-x (PMC6444439; doi:10.1186/s13148-019-0637-x)
Supplement: Supplementary file 5 — Figure S3. Correlation heatmap of methylation levels inside the two most significant DMR of folate and alcohol. (PDF 43 kb) [file 13148_2019_637_MOESM5_ESM.pdf]

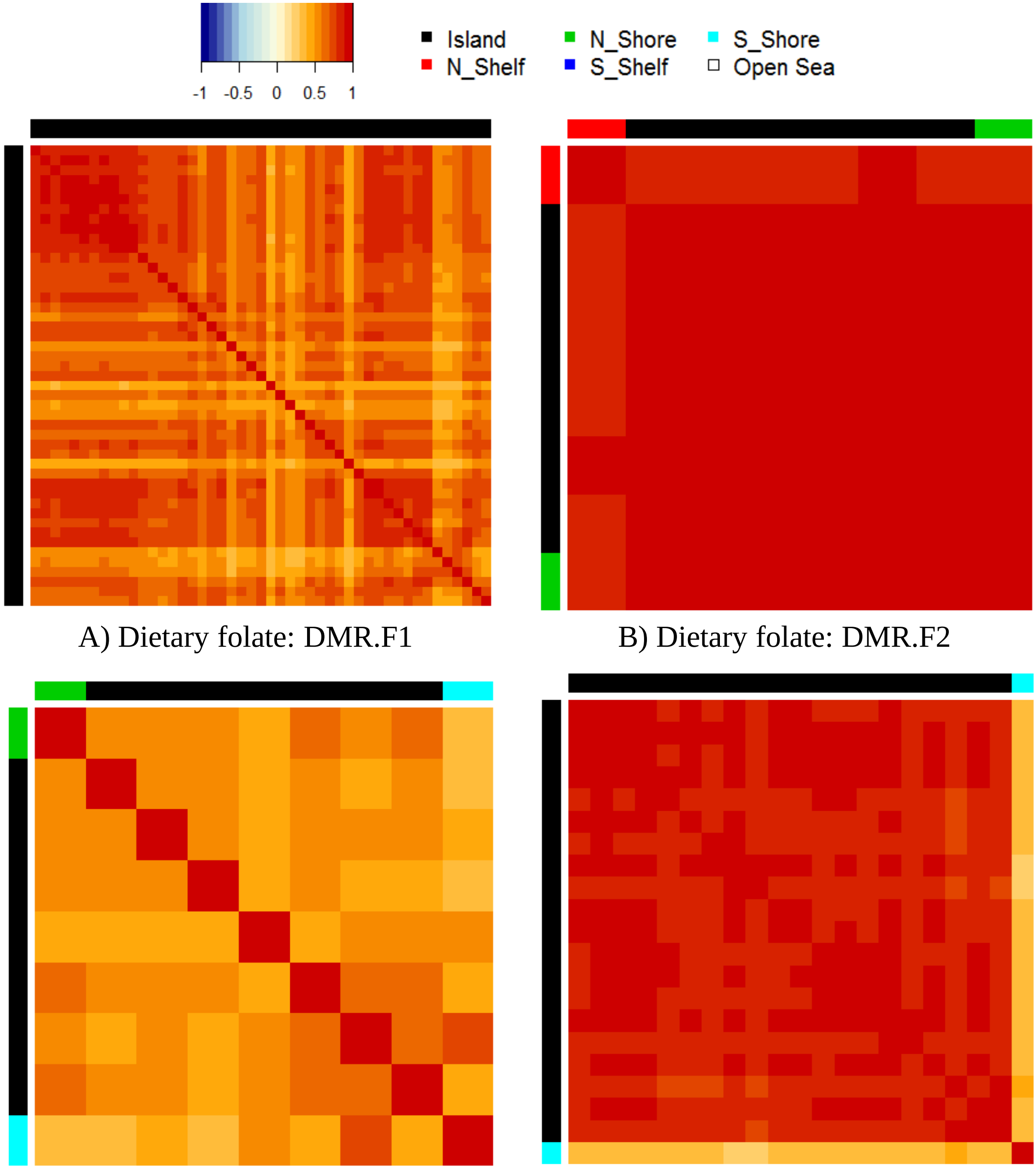

A) Dietary folate: DMR.F1

B) Dietary folate: DMR.F2

C) Alcohol intake: DMR.A1

D) Alcohol intake: DMR.A2
